# Breast Milk from Non-Obese Women with a High Omega-6 to Omega-3 Fatty Acid Ratio, but Not from Women with Obesity, Increases Lipogenic Gene Expression in 3T3-L1 Preadipocytes, Suggesting Adipocyte Dysfunction

**DOI:** 10.3390/biomedicines10051129

**Published:** 2022-05-13

**Authors:** Peter Isesele, Samantha Enstad, Pham Huong, Raymond Thomas, Carol L. Wagner, Sarbattama Sen, Sukhinder K. Cheema

**Affiliations:** 1 Department of Biochemistry, Memorial University, St. John’s, NL A1C 5S7, Canada; poisesele@mun.ca; 2 Winnie Palmer Hospital for Women and Babies, Orlando, FL 32806, USA; samantha.enstad@orlandohealth.com; 3 School of Science/Boreal Ecosystems and Agriculture Sciences, Memorial University, Corner Brook, NL A2H 5G4, Canada; tpham@grenfell.mun.ca (P.H.); rthomas@grenfell.mun.ca (R.T.); 4 Department of Pediatrics, Division of Neonatology, Shawn Jenkins Children’s Hospital, Medical University of South Carolina, Charleston, SC 29425, USA; wagnercl@musc.edu; 5 Department of Pediatric Newborn Medicine, Brigham and Women’s Hospital, Harvard Medical School, Boston, MA 02115, USA; ssen2@bwh.harvard.edu

**Keywords:** adipogenesis, lipogenesis, breast milk, obesity, polyunsaturated fatty acids, 3T3-L1 preadipocytes

## Abstract

Maternal body mass index is associated with breast milk (BM) fatty acid composition. This study investigated the effects of BM omega (n)-6:n-3 polyunsaturated fatty acids (PUFAs) from non-obese women and women with obesity on the process of adipogenesis in 3T3-L1 preadipocytes. BM samples were collected from non-obese women (BMNO) and women with obesity (BMO) at one month postpartum. The fatty acid composition was measured, and BMNO and BMO groups with the lowest (Q1) and highest (Q4) quartiles of n-6:n-3 PUFA ratios were identified. 3T3-L1 preadipocytes were differentiated in the presence or absence of BM. Lipid accumulation and the expression of genes involved in lipogenesis and lipolysis were measured. Treatment with BMNO containing high (vs. low) n-6:n-3 PUFA ratios significantly increased the mRNA expression of lipogenic genes (acetyl-CoA carboxylase, fatty acid synthase, and stearoyl-CoA desaturase); however, there was no effect when cells were treated with BMO (with either low or high n-6:n-3 PUFA ratios). Treatment with BMO (high n-6:n-3 PUFA ratio) caused larger lipid droplets. Our findings demonstrated that BMNO with a high n-6:n-3 PUFA ratio was associated with a higher expression of lipogenic genes, while BMO with a high n-6:n-3 PUFA ratio showed larger lipid droplets, suggesting adipocyte dysfunction. These findings may have implications in the BM-mediated programming of childhood obesity.

## 1. Introduction

Obesity has become a global epidemic [1]. Women with pre-pregnancy obesity are at an increased risk for adverse maternal and perinatal outcomes [2,3]. Both obesity and excessive weight gain during pregnancy are risk factors for childhood adiposity and later cardiovascular and respiratory morbidity of the offspring [4]. Diet plays a key role in the regulation of an infant’s growth and development. Exclusive breastfeeding is recommended for infants in the first six months of life because breast milk (BM) is thought to contain the ideal nutrient composition as well as non-nutritive benefits [5,6].

The fatty acid composition of BM is altered depending on the type of dietary fats consumed in the maternal diet [7]. For example, the levels of essential polyunsaturated fatty acids (PUFAs), linoleic acid (LA), and α-linolenic acid (ALA) vary with the maternal dietary intake of these fatty acids. Omega (n)-6 PUFAs are generally linked to higher inflammation because the conversion of LA to arachidonic acid (AA) provides a substrate for potent pro-inflammatory mediators, including prostaglandins and leukotrienes [8]. On the other hand, n-3 PUFAs are often categorized as less inflammatory in nature due to their inhibitory effects on the secretion of pro-inflammatory mediators, reduction in macrophage migration into the adipose tissue, and production of anti-inflammatory eicosanoids [9]. Thus, the ratio of n-6 to n-3 PUFAs is important in maintaining inflammatory balance. Moreover, the ratio of n-6:n-3 PUFAs plays an important role in the maintenance of lipid metabolism and oxidative stress [10].

We previously showed that BM with a higher n-6 PUFA content contained a higher concentration of soluble pro-inflammatory cytokines in healthy women from Newfoundland and Labrador and induced events similar to insulin resistance in 3T3-L1 adipocytes [11]. We and others have also previously shown that BM from women with obesity (BMO) has a higher n-6:n-3 PUFA ratio and increased inflammatory cytokine and leptin levels that are associated with higher infancy weight gain and childhood obesity [12,13,14,15]. Higher infant weight gain may be a consequence of increased fat deposition.

Both n-6 and n-3 PUFAs act as ligands to regulate the expression of genes involved in lipogenesis [16]. However, the mechanisms by which a high n-6:n-3 PUFA ratio in BM may contribute to higher weight gain in infants are not known. The objective of this study was to delineate the mechanisms underlying the lactational programming of infant growth. We hypothesized that the differentiation of 3T3-L1 preadipocytes in the presence of BMO, and BM with a high n-6:n-3 PUFA ratio, may increase lipogenesis and induce adipocyte dysfunction in the 3T3-L1 cells, which could be responsible for the higher infancy weight gain and childhood obesity observed in our previous study [12].

## 2. Materials and Methods

### 2.1. Ethics Statement

This study was approved by the Health Research Ethics Board (2017.231) of the Memorial University of Newfoundland and Labrador, Canada and the Partners Institutional Review Board. The parent study under which these samples were collected was approved by the Medical University of South Carolina Institutional Review Board. Informed consent was obtained from all subjects involved in this study. Participants were selected from a randomized controlled trial of maternal vitamin D supplementation during lactation (registered via ClinicalTrials.gov (accessed on 9 March 2022) NCT00412074): a CONSORT flow diagram of participant selection is given in Supplementary Figure S1.

### 2.2. Collection of BM Samples

Participants were selected from a randomized controlled trial of maternal vitamin D supplementation [17]. In the present study, participants were included if they were in the control (placebo) group, if they had an available maternal body mass index (BMI) value at one month postpartum, and if they had BM archived at one month postpartum. 

Participants were categorized based on BMI into two groups, where BMI < 30 kg/m^2^ was categorized as non-obese/overweight (*n* = 20) and ≥30 kg/m^2^ as obese (*n* = 20). BM samples (mature milk) were collected from the participants at one-month postpartum using a hospital-grade electric pump in the morning. Participants were asked to collect a complete feed from the opposite breast from which the infant was feeding. BM samples (with no linkage to personal identifiers) were shipped frozen to Memorial University, Newfoundland and immediately stored at −80 °C until analysis.

### 2.3. Preparation of BM Whey

BM samples from both non-obese women (BMNO) and women with obesity (BMO) were grouped into quartiles, based on the n-6:n-3 PUFA concentrations. The n-6 PUFAs included the fatty acids C18:2n6, C18:3n6, and C20:4n6, while the n-3 PUFAs included the fatty acids C18:3n3, C20:3n3, C20:4n3, C20:5n3, C22:5n3, and C22:6n3. BM whey was separated from whole BM according to the method previously described in [18] with modifications [11]. Briefly, whole BM was thawed on ice and centrifuged at 500× *g* for 25 min at 4 °C to remove cellular components. The supernatants were then centrifuged at 13,700× *g* for 10 min at 4 °C to separate the whey from the lipid layer. Aqueous whey (clear phase) was pooled based on the n-6:n-3 PUFA ratios (low and high) separately for the non-obese and obese women, divided into aliquots to avoid repeated freeze–thaw cycles, and stored at −80 °C until needed for further analysis.

### 2.4. Fatty Acid Analysis of BM Samples

The fatty acid analysis of BMNO and BMO samples was performed using GC-MS/FID analysis as previously described in [19]. Briefly, using a BPX70 high-resolution column (10 m 0.1 mm ID 0.2 m; Canadian Life Science, Peterborough, ON, Canada) and helium as the carrier gas at a flow rate of 1 mL/min, methylated fatty acids were separated. Commercial standards (Supelco 37 component mix, Supelco PUFA No. 3, Supelco FAME mix C8–C24; Sigma Aldrich, Oakville, ON, Canada) and the NIST database were used to determine the methylated fatty acids (ThermoScientific, Burlington, ON, Canada). Individual fatty acid quantities were estimated using standard curves constructed from the standard mixtures, and the values were expressed as a nanomolar percentage of total fatty acid (nmol percent) for each BM sample. The ratio of n-6 to n-3 PUFAs was calculated by first taking a sum of the concentrations of the n-6 PUFAs (C18:2n6, C18:3n6, and C20:4n6) and n-3 PUFAs (C18:3n3, C20:3n3, C20:4n3, C20:5n3, C22:5n3, and C22:6n3) of each sample for both non-obese and obese groups; the ratio of total n-6 PUFAs to total n-3 PUFAs (n-6:n-3 PUFA ratio) was then calculated for each sample for both the non-obese and obese groups. Based on the n-6:n-3 PUFA ratios, the 20 samples in the obese and the non-obese groups were each grouped into quartiles (low (Q1) and high (Q4)). 

### 2.5. Treatment of 3T3-L1 Preadipocytes with BM

The 3T3-L1 cells were obtained from the American Type Culture Collection (ATCC # CL-173, Manassas, VA, USA). Insulin, isobutyl-methylxanthine (IBMX), dexamethasone (Dex), and Dulbecco’s modified Eagle’s medium (DMEM) were obtained from Sigma Aldrich (Oakville, ON, Canada). Fetal bovine serum (FBS), antibiotic-antimycotic (100×), and newborn calf serum (NBCS) were obtained from Gibco Life Technologies (Burlington, ON, Canada). Preadipocytes (3T3-L1) were grown to 70% confluency and were induced to differentiate by adding a differentiation cocktail (insulin (10 μg/mL), 1 μM Dex, and 0.5 mM IMBX) in DMEM containing 10% FBS along with 1% BM whey (both Q1 and Q4 from BMNO and BMO), as per previous publications [11,20,21,22]. Control cells received only the differentiation cocktail. After 48 h, the media were changed to DMEM containing 10% FBS and insulin (10 μg/mL) along with 1% BM whey (Q1 and Q4) from both the BMNO and BMO groups. The media were replaced with DMEM containing 10% FBS + 1% BM whey every 48 h till day eight, when the cells were fully differentiated and harvested. The control cells received similar media at each stage of differentiation without BM whey. 

### 2.6. Oil Red O Staining

Lipid accumulation in 3T3-L1 mature adipocytes was measured using an oil red O solution in 0.5% isopropanol (Millipore, Burlington, ON, Canada). After eight days of differentiation, the cells were washed thrice with 1X phosphate-buffered saline (PBS), and 1 mL of oil red O dye was added; the cells were incubated for 15 min at room temperature. The dye was removed, and the cells were washed with 2 mL of distilled water to remove the non-binding dye. Stained adipocytes were viewed using a Leica DMIL LED Tissue Culture Microscope (Leica Microsystems Inc., Richmond Hill, ON, Canada), and images (3 per well) were taken using the Infinity Camera Analyze Software (Lumenera Corporation, Ottawa, ON, Canada) at 400× magnification. Stained lipids were eluted by adding 500 µL of 100% isopropanol to each well. The absorbance of the extracted dye was read at 520 nm; isopropanol was used as a blank. The size of lipid droplets was measured using the ImageJ software (Version 1.8; NIH, Bethesda, MD, USA).

### 2.7. RNA Extraction and Real-Time Quantitative Polymerase Chain Reaction (qPCR)

Total RNA was extracted from mature 3T3-L1 adipocytes on day 8 of differentiation using TRIzol [23]. The primers used for qPCR were designed using NCBI primer blast (www.ncbi.nlm.nih.gov/tools/primer-blast; accessed on 1 October 2018) and were obtained from IDT Technologies (Coralville, IA, USA). Primer sequences are given in Supplementary Table S1. Amplification was performed using iQ SYBR Green Supermix (#1708880, Bio-Rad, Hercules, CA, USA) with a reaction volume of 10 µL and 50 ng cDNA per reaction. Samples were run for 40 cycles (denaturation at 95 °C for 15 mins, annealing at 58–60 °C for 15 s, and extension at 72 °C for 15 s) using the CFX96^TM^ Real-Time System. Data were analyzed using the CFX Manager^TM^ Software Version 3.0. The delta Ct values for each gene of interest were recorded, and the gene expression was normalized against RPLP0 (large ribosomal protein) as the housekeeping gene. The expression levels were measured using the ΔΔCt method [24]. 

### 2.8. Statistical Analysis

Individual fatty acids were compared between BMNO and BMO using a Student’s *t*-test. The comparison between the Q1 and Q4 of BMNO and BMO was carried out using two-way ANOVA. Gene expression data were analyzed between quartiles within BMNO and BMO and between similar quartiles of BMNO and BMO using two-way ANOVA followed by Bonferroni post hoc multiple comparisons to compare the main effects of quartiles, maternal status, and the interactions between them. For all experiments, the results were presented as mean ± standard deviation (SD), *n* = 3, and *p* < 0.05 was considered significant. Data were analyzed using the Graph Pad Prism Software (version 8). 

## 3. Results

### 3.1. Participant Characteristics

The descriptive characteristics of the non-obese and obese women in the study population are presented in Table 1. The mean BMI of the non-obese women was 25 kg/m^2^, while the women with obesity had a mean BMI of 33 kg/m^2^. There was no substantial difference in ethnicity between non-obese women and women with obesity.

### 3.2. Effects of Maternal Obesity on the Fatty Acid Composition of BM

The fatty acid composition of BMNO and BMO is presented in Table 2. There was no significant difference in the total saturated fatty acids (SFAs), C14:0 and C16:0, between BMNO and BMO; however, BMNO had higher C18:0 (*p* < 0.05) compared to BMO. There was no significant difference in the total monounsaturated fatty acids (MUFAs) and C16:1 between BMNO and BMO; however, BMNO had higher C18:1 (*p* < 0.05) compared to BMO. There were no differences in AA (C20:4n6) levels between BMNO and BMO; however, BMO had significantly (*p* < 0.01) higher LA (C18:2n6) and total n-6 PUFAs (*p* < 0.05), compared to BMNO. There was no difference in docosahexaenoic acid (DHA), eicosapentaenoic acid (EPA), and total n-3 PUFAs between BMNO and BMO; however, BMO had a higher (*p* < 0.05) n-6:n-3 PUFA ratio than that of BMNO (BMO 9.2 ± 1.56 vs. BMNO 8.0 ± 1.38; *p* = 0.01).The fatty acid composition of BM based on the quartile (Q1 and Q4) is presented in Table 3, while Q1, Q2, Q3, and Q4 are presented in the Supplementary Table S2. BMNO Q4 and BMO Q4 had n-6:n-3 PUFA ratios of 9.67 and 11.35, respectively, whereas BMNO Q1 and BMO Q1 had n-6:n-3 PUFA ratios of 6.77 and 7.63, respectively.

### 3.3. Maternal BMI and BM n-6:n-3 PUFA Ratio Affects Lipid Accumulation in Mature 3T3-L1 Adipocytes

**Figure 1:**
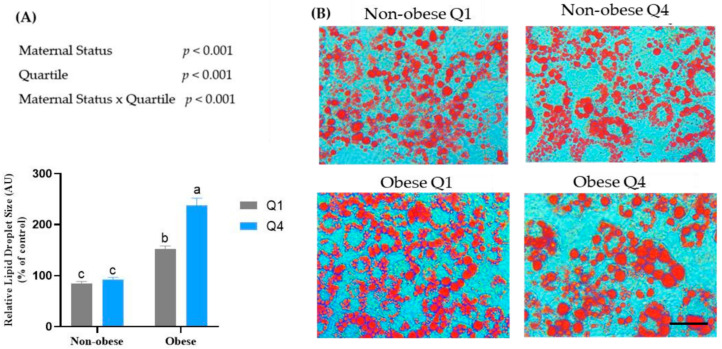
Effects of breast milk on lipid accumulation in 3T3-L1 adipocytes. Preadipocytes were differentiated for eight days in the presence or absence of breast milk (BM) whey, and oil red O staining was performed on day 8. Stained adipocytes were viewed using a Leica DMIL LED Tissue Culture Microscope, and images were taken using the Infinity Camera Analyze Software at 400× magnification. Scale bar = 50 µm (**A**) Relative lipid droplet size analyzed using the ImageJ software. Values are expressed as mean ± SD, *n* = 3, different superscripts (a, b, c) are used to denote significant differences between the treatment groups, *p* < 0.05 was considered significant (**B**) Representative images of the cells stained with oil red O. Q—quartile.

The cells treated with BMO had a larger lipid droplet size compared to cells treated with BMNO (Figure 1A,B). In addition, the cells treated with BMO Q4 displayed a larger lipid droplet size (1289.1 ± 422 µm^2^, *p* < 0.001) compared to the cells treated with BMO Q1 (805.5 ± 158.1 µm^2^, *p* < 0.01), BMNO Q1 (485.7 ± 131.6 µm^2^, *p* < 0.001), and BMNO Q4 (450.37 ± 95; *p* < 0.001). However, there was no difference in lipid droplet size in cells treated with BMNO Q4 vs. Q1.

### 3.4. Maternal BMI and BM n-6:n-3 PUFA Ratio Alters the mRNA Expression of Lipogenic Genes

**Figure 2:**
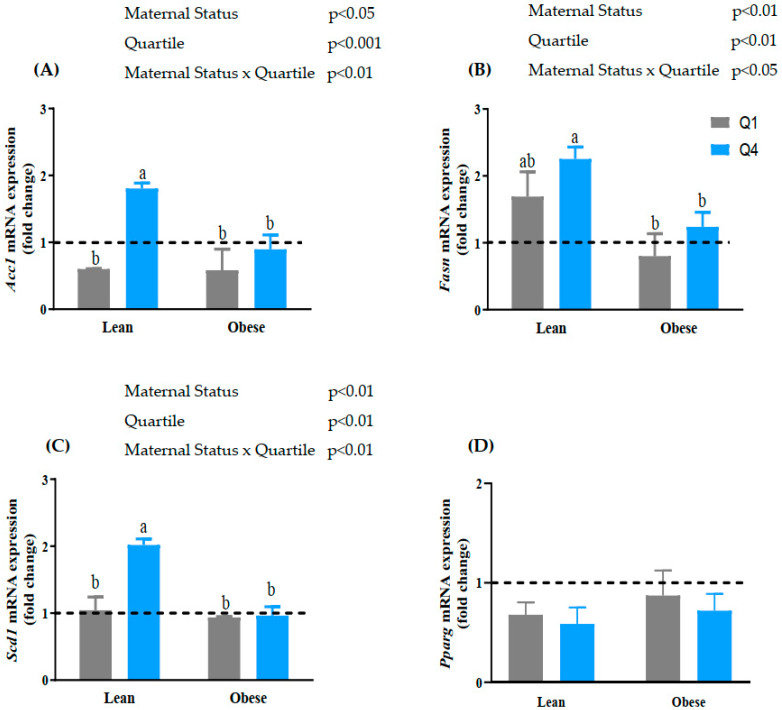
Effects of non-obese and obese breast milk with high and low n-6:n-3 PUFA ratios on the mRNA expression of lipogenic and adipogenic genes in 3T3-L1 adipocytes. Preadipocytes were differentiated for 8 days in the presence or absence of breast milk (BM) whey. Data represent the mRNA expression of: (**A**) acetyl CoA carboxylase (*Acc1*), (**B**) fatty acid synthase (*Fasn*), (**C**) stearoyl-CoA desaturase-1 (*Scd1)*, and (**D**) peroxisome proliferator-activated receptor gamma (*Pparg*). The mRNA expression was normalized with RPLP0 as the reference gene. Values for the control cells (no breastmilk treatment) were set to 1.0 and are represented by the dashed line. Values are expressed as mean ± SD, *n* = 3. Data were analyzed using two-way ANOVA to determine the effect of treatment, and a Bonferroni post hoc test was used to determine differences when there was a significant interaction. Different superscript (a, b) represent significant differences between treatment groups, *p* < 0.05 was considered significant. Q—quartile.

The initial step of lipogenesis involves the conversion of acetyl-CoA to malonyl-CoA by *Acc1*, which is the rate-limiting step in *de novo* fatty acid synthesis. The mRNA Expression of *Acc1* was higher in the cells treated with BMNO compared to the cells treated with BMO (+68% increase; *p* < 0.01; Figure 2A). There was a significant interaction between maternal BMI status and quartile (interaction, *p* < 0.05). Cells treated with BMNO Q4 had a higher mRNA expression of *Acc1* compared to cells treated with BMNO (Q1). Furthermore, BMNO Q4-treated cells had a higher mRNA expression of *Acc1* compared to BMO Q4-treated cells. There was no change in *Acc1* mRNA expression in BMO Q1- and BMO Q4-treated cells. 

Malonyl-CoA synthesized by Acc1 is converted to palmitate (C16:0) by *Fasn*. There was a significant effect of maternal BMI status on the mRNA expression of *Fasn*; cells treated with BMNO had a higher expression compared to the cells treated with BMO (*p* < 0.01; Figure 2B). There was also a significant interaction between maternal status and quartile (interaction *p* < 0.05). There was no significant difference in *Fasn* mRNA expression between cells treated with Q4 BMNO vs. Q1 BMNO and BMO Q4 vs. BMO Q1. 

Synthesized palmitate and stearic acid (C18:0) undergo desaturation by *Scd1* activity to generate palmitoleic acid (C16:1) and oleic acid (C18:1), respectively. Oleic acid is considered as the storage fatty acid. There was a significant difference in the mRNA expression of *Scd1* between cells treated with BMO and BMNO, revealing a higher expression in the cells treated with BMNO compared to the cells treated with BMO (*p* < 0.01; Figure 2C). Furthermore, there was a significant interaction between maternal BMI status and quartile (interaction, *p* < 0.05). Cells treated with Q4 BMNO had a higher mRNA expression of *Scd1* compared to cells treated with Q1 BMNO (+100%; *p* < 0.01, Figure 2C). There was no significant difference between BMO Q4- and BMO Q1-treated cells. Interestingly, there was no effect of maternal BMI or the n-6:n-3 PUFA ratio quartile on the expression of *Pparg* (Figure 2D).

### 3.5. Maternal BMI and BM n-6:n-3 PUFA Ratio Had No Significant Effect on the mRNA Expression of Lipolytic Genes

**Figure 3:**
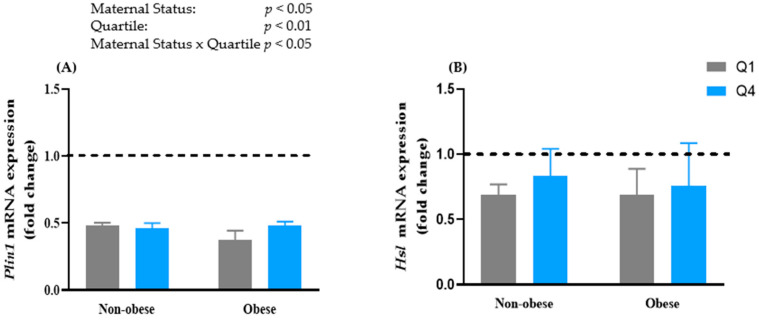

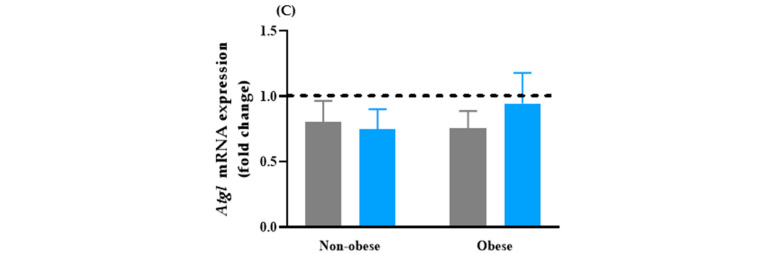
Effects of non-obese and obese breast milk with high and low n-6:n-3 PUFA ratios on the mRNA expression of lipolytic genes in 3T3-L1 adipocytes. Preadipocytes were differentiated for 8 days in the presence or absence of breast milk (BM) whey. Data represent the mRNA expression of: (**A**) perilipin (*Plin1*), (**B**) adipose triglyceride lipase (*Atgl*), and (**C**) hormone-sensitive lipase (*Hsl*). The gene expression was normalized with RPLP0 as the housekeeping gene. Values for the control cells (no breastmilk treatment) were set to 1.0 and are represented by the dashed line. Values are expressed as mean ± SD, *n* = 3. Data were analyzed using two-way ANOVA to determine the effect of treatment, and a Bonferroni post hoc test was used to determine differences when there was a significant interaction. *p* < 0.05 was considered significant. Q—quartile.

Perilipin (*Plin1*) is a lipid droplet-associated protein that is an integral component of lipolysis [25]. There was no significant difference in the mRNA expression of *Plin1* between cells treated with BMO and BMNO (Figure 3A). Similarly, the n-6:n-3 PUFA ratio had no effect on *Plin1 mRNA* expression. Triacylglycerols are hydrolyzed through the process of lipolysis, which is regulated by *Atgl* and *Hsl.* There was no significant difference in the mRNA expression of *Atgl* between cells treated with BMNO and BMO compared to control cells (Figure 3B). Similarly, there was no significant difference between cells treated with BMO Q4 and those treated with BMO Q1. There was also no significant difference in the mRNA expression of *Hsl* between cells treated with BMNO and BMO (Figure 3C). Similarly, there was no significant difference in the mRNA expression of *Hsl* between cells treated with BMO Q4 and BMO Q1 (Figure 3C).

## 4. Discussion

Exclusive breastfeeding is recommended for at least six months due to its durable benefits for maternal and child health. Recently, differences in BM composition, particularly fatty acids, across a range of maternal metabolic conditions have been reported [26,27,28]. Our group has recently shown that infants born to women with obesity have an accelerated BMI and adiposity accrual compared to that of infants born to non-obese women [12]. We and others have reported an association between infant growth and BM n-6:n-3 PUFA content [12,28]. However, the mechanisms by which BM fatty acid composition affects infant BMI have not been explored. Here, we sought to investigate the role of maternal BMI and BM n-6:n-3 PUFA ratio on adipocyte function and their effect on the development of obesity. Our findings demonstrated that the BM n-6:n-3 PUFA ratio had a dominant effect on upregulating the expression of lipogenic genes compared to maternal BMI.

The fat composition of BM is variable [29] and it reflects changes in the maternal diet [30], particularly in the consumption of long-chain PUFAs. We found that BMO had higher levels of LA (C18:2n6) compared to BMNO. LA is an essential n-6 PUFA and is the main fatty acid found in a typical Western-style diet [8]. A significant positive correlation has been found between LA intake and LA levels in BM [31]. LA is the precursor for AA; the conversion of LA to AA occurs by sequential ∆-6 desaturation, elongation, and ∆-5 desaturase reactions [32]. We did not find differences in the AA levels in BMNO and BMO, suggesting that maternal obesity has minimal effects on AA levels. Studies have shown that obesity is associated with increased delta-6 desaturase activity [33,34]. We found that BMO had a higher delta-6 desaturase index (GLA:LA) compared to BMNO, suggesting that the higher levels of LA in BMO are likely due to a higher dietary intake of LA. There was no difference in DHA, EPA, and total n-3 PUFAs between BMNO and BMO; however, BMO had a higher n-6:n-3 PUFA ratio compared to BMNO, suggesting that the difference in the n-6:n-3 PUFA ratio was due to higher levels of n-6 PUFAs in the BMO group. DHA contributed less to the n-6:n-3 PUFA ratio. Higher levels of DHA are generally observed among coastal populations due to their higher consumption of seafood. The percentage of DHA in BMNO and BMO was 0.46% and 0.41%, respectively, which is higher than the worldwide mean DHA levels of 0.32% in BM [35]. The geographical location of the majority of the study participants may have contributed to the higher levels of DHA observed in our study, thereby leading to the lack of differences in DHA levels in these groups. The n-6:n-3 PUFA ratio in BM has been shown to be associated with a number of other diseases, such as an increased risk of allergic manifestations [36], inflammation, and insulin resistance [11] and increased infant fat mass [37]. However, the mechanisms by which BM fatty acids influence adipose tissue function is still under-investigated.

Lipid accumulation in adipose tissue occurs through the sequential expression of genes involved in triacylglycerol synthesis [38,39]. The increased expression of *Acc1* in BMNO Q4 implies a higher conversion of acetyl-CoA to malonyl-CoA, which is known to inhibit carnitine palmitoyltransferase (CPT1), the key enzyme regulating β-oxidation. Thus, the upregulation of *Acc1* may reduce β-oxidation and increase lipid synthesis. The concurrent increase in *Fasn* and *Scd1* mRNA expression by BMNO Q4 further suggests an increase in the endogenous synthesis of fatty acids. Increased lipogenesis may be the potential mechanism by which high BM n-6:n-3 PUFA ratios are associated with infant adiposity, as we previously reported [12]. The increase in lipogenic gene expression promoted by a high BM n-6:n-3 PUFA ratio is similar to our previous study, where BM from healthy women from Newfoundland and Labrador with a high n-6:n-3 PUFA ratio upregulated *Scd1* mRNA expression [11]. The increased expression of *Scd1* is directly associated with insulin resistance [40,41,42] and fat deposition [43]. Despite an increase in the mRNA expression of lipogenic genes with BM from non-obese women with high n-6:n-3 PUFA ratios, there was no effect on the mRNA expression of *Pparg,* suggesting that the regulation of lipogenic genes is likely independent of *Pparg*. 

Interestingly, there was no effect of BMO (Q1 and Q4) on the mRNA expression of lipogenic genes. However, we observed a significantly larger lipid droplet size in cells treated with BMO (both Q1 and Q4). The larger lipid droplet size observed in the cells treated with BMO could possibly result from pathways independent of lipogenesis, such as adipocyte dysfunction. A larger droplet size with enlarged adipocytes is characteristic of insulin resistance and adipose dysfunction [44,45]. The reason for the absence of an effect on lipogenic gene expression by BMO with a high n-6:n-3 PUFA ratio could be that the large lipid droplets limit the expansion of triacylglycerol storage. It has been suggested that large adipocytes may downregulate lipogenic genes to limit the expansion of triacylglycerol stores [46]. Macrophage migration inhibitory factor (*Mif*) is positively associated with adipocyte size and negatively associated with insulin action [47]. We measured the mRNA expression of *Mif* (Supplementary Figure S2) and found that the cells treated with BMO Q4 had higher expression compared to those treated with BMO Q1. Further studies are required to confirm an association between *Mif* expression, lipid droplet size, and insulin resistance in cells treated with BM. Overall, our findings demonstrate that the BM n-6:n-3 PUFA ratio has a dominant effect on upregulating the expression of lipogenic genes compared to maternal BMI. 

Both BMNO- and BMO-treated cells showed no effect on the mRNA expression of *Plin1*, suggesting that the treatment had no effect on lipolysis. Despite the larger lipid droplet size in BMO Q4-treated cells compared to BMO Q1-treated cells, there was no difference in the expression of *Atgl* and *Hsl,* which are the dominant lipolytic genes. These findings suggest that the increased lipid droplet size was not a result of decreased triacylglycerol hydrolysis. In addition to BM fatty acid composition, other factors, such as higher levels of oxidative stress, have also been suggested to affect lipogenesis. MDA is a major secondary product of lipid peroxidation [18,48,49] and is known to suppress lipogenesis [50]. We observed higher levels of MDA in BMO- (2.82 ± 0.91 µM) compared to BMNO (2.11 ± 0.56 µM; Supplementary Figure S3); thus, the high levels of MDA in BMO may also have induced adipose dysfunction. Furthermore, it has been shown that there is a high correlation between leptin and MDA levels in the blood of obese individuals [51], along with an association between leptin and oxidative stress [52,53,54]. We previously reported that BMO had elevated levels of leptin (1414.2 ± 1185 pg/mL) compared to BMNO (398.85 ± 255.59 pg/mL) in the same cohort used in the current study [12]. It has been suggested that increased oxidative stress, as demonstrated by the high levels of MDA in BMO, may be regulated by hyperleptinemia [52]. The higher levels of lipid peroxides in BMO could potentially affect the health of the newborn. We previously reported that the BMO used in this study had higher levels of interleukin (IL)-1β (1.24 ± 1.14 pg/mL) compared to BMNO (0.57 ± 0.42 pg/mL) [12]. Similarly, higher levels of IL-6 were observed in BMO (2.36 ± 1.92 pg/mL) compared to BMNO (1.09 ± 0.70 pg/mL) [12]. IL-1β and IL-6 have been shown to induce insulin resistance in adipocytes [55,56]. Leptin has been shown to induce fat oxidation and downregulate the expression of genes involved in lipogenesis [57,58,59,60]. Thus, high levels of leptin and pro-inflammatory cytokines in BM, as reported previously [12], may also influence the regulation of lipogenic genes and insulin sensitivity. We previously reported that BM proinflammatory cytokines (measured in the whey portion of BM) were positively correlated with the BM n-6:n-3 PUFA ratio [11]. A similar study previously reported that the lipid portion of BM promotes adipogenesis [21]. In future studies, we will examine the role of the lipid component of BM in adipogenesis.

## 5. Conclusions

**Figure 4:**
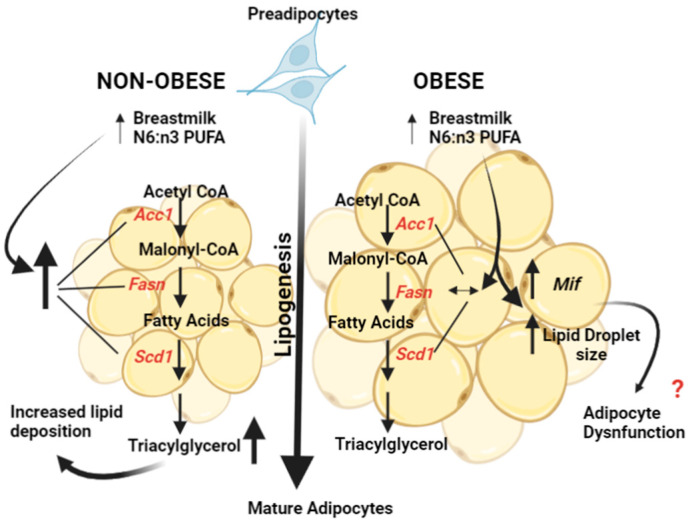
Proposed mechanisms by which breast milk composition affects adipocyte function. Breast milk (BM) of non-obese women with a high omega n-6:n-3 PUFA ratio increased the mRNA expression of lipogenic genes, whereas BM of women with obesity resulted in a larger lipid droplet size, suggesting adipocyte dysfunction. *Acc1*—acetyl CoA carboxylase; *Fasn*—fatty acid synthase; *Mif*—macrophage migration inhibitory factor; *Scd1*—stearoyl-CoA desaturase-1.

Our findings demonstrate that the BM n-6:n-3 PUFA ratio has a dominant effect on upregulating the expression of lipogenic genes compared to maternal BMI. Furthermore, the effects of BM on adipocyte function appear to be independent of *Pparg* mRNA expression and lipolysis. Although BMO had no significant effect on lipogenesis, it caused a larger lipid droplet size, suggesting adipocyte dysfunction. Figure 4 provides a potential mechanism by which BMO and BMNO regulate adipocyte function. Our findings provide potential preliminary mechanistic insight into how BM n-6:n-3 PUFA ratios are associated with adiposity. These findings may have implications for the effects of BM composition on regulating adiposity in newborns and their future health.

## Figures and Tables

**Table 1:** Demographics of the study participants.

	Non-Obese	Obese
N	20	20
Age (years), mean (SD)	31.5 (6.2)	29 (7)
BMI (kg/m^2^), mean (SD)	25 (2.5)	33 (3.7)
**Race/Ethnicity, *n* (%)**		
Caucasian, non-Hispanic	15 (75%)	9 (45%)
Hispanic	3 (15%)	7 (35%)
African American, non-Hispanic	0	4 (20%)
Asian or Pacific Islander	2 (10%)	0
**Education Level *n* (%)**		
High school		
Some college	3 (15%)	3 (15%)
Associate degree (2 years college)	1 (5%)	0
Bachelor’s degree	3 (15%)	5 (25%)
Master’s degree	7 (35%)	3 (15%)
Ph.D./Sc.D./M.D.	1 (5%)	1 (5%)

Education level is the highest level of education achieved. BMI—body mass index.

**Table 2:** The fatty acid composition of breast milk from non-obese women and women with obesity (obese).

Fatty Acid Name	FA (nmol%)	Non-Obese	Obese	*p*-Value
Palmitic acid	C16:0	22.57 ± 2.45	21.41 ± 2.96	*p* = 0.104
Stearic acid	C18:0	6.77 ± 1.08 ^a^	5.62 ±0.99 ^b^	*p* < 0.0001
Palmitoleic acid	C16:1	2.55 ± 0.74	2.49 ± 0.76	*p* = 0.669
Oleic acid	C18:1	33.02 ± 3.19 ^a^	30.46 ± 3.96 ^b^	*p* < 0.001
Eicosenoic acid	C20:1n9	0.35 ± 0.06	0.33 ± 0.06	*p* = 0.141
Linoleic acid (LA)	C18:2n6	22.98 ± 4.55 ^a^	27.10 ± 4.23 ^b^	*p* < 0.0001
γ-Linolenic acid (GLA)	C18:3n6	0.26 ± 0.04	0.28 ± 0.06	*p* = 0.462
Eicosatrienoic acid	C20:3n6	0.72 ± 0.09	0.83 ± 0.19	*p* = 0.023
Arachidonic acid (AA)	C20:4n6	0.75 ± 0.11	0.79 ± 0.12	*p* = 0.573
α-Linolenic acid (ALA)	C18:3n3	1.71 ± 0.53	1.81 ± 0.46	*p* = 0.777
Eicosatrienoic acid	C20:3n3	0.19 ± 0.04	0.20 ± 0.04	*p* = 0.886
Eicosatetraenoic acid	C20:4n3	0.16 ± 0.02	0.16 ± 0.02	*p* = 0.784
Eicosapentaenoic acid (EPA)	C20:5n3	0.23 ± 0.06	0.22 ± 0.05	*p* = 0.420
Docosapentaenoic acid	C22:5n3	0.34 ± 0.06	0.36 ± 0.08	*p* = 0.709
Docosahexaenoic acid (DHA)	C22:6n3	0.46 ± 0.11	0.41 ± 0.12	*p* = 0.161
	ΣSFA	36.27 ± 3.77	34.56 ± 4.66	*p* = 0.072
	ΣMUFA	35.92 ± 3.12	33.28 ± 6.68	*p* = 0.510
	ΣPUFA	27.81 ± 5.04 ^b^	32.16 ± 4.52 ^a^	*p* < 0.0001
	Σn6-PUFA	24.70 ± 4.58 ^b^	29.00 ± 4.24 ^a^	*p* = 0.037
	Σn3-PUFA	3.11 ± 0.59	3.17 ± 0.49	*p* = 0.937
	n-6:n-3 PUFA	7.95 ± 1.38 ^a^	9.15 ± 1.56 ^b^	*p* < 0.0001
	D6D	0.01 ± 0.01 ^b^	0.13 ± 0.01 ^a^	*p* < 0.001
	D5D	1.04 ± 0.01	0.05 ± 0.63	*p* = 0.159

Data are expressed as nmol% of the total extracted fatty acids; values are expressed as mean ± SD, *n* = 17–20. Data were analyzed by Student’s *t*-test, and different superscripts (a, b) are used to denote significant differences between the treatment groups. D6D—delta-6 desaturase (GLA/LA); D5D—delta-5 desaturase (C20:4n6/C20:3n6); FA—fatty acids; ΣSFA—sum of saturated fatty acids; ΣMUFA—sum of monounsaturated fatty acids; ΣPUFA—sum of polyunsaturated fatty acids; Σn-6—sum of omega-6 polyunsaturated fatty acids; Σn-3—sum of omega-3 polyunsaturated fatty acids. *p* < 0.05 was considered significant.

**Table 3:** The fatty acid composition of breast milk by quartiles with low and high n-6:n-3 PUFA ratios.

		**Non-Obese**		**Obese**		
**Fatty Acid Name**	**FA (nmol%)**	**Q1**	**Q2**	**Q3**	**Q4**	***p*-Value**
Myristic acid	C14:0	8.00 ± 1.74	5.96 ± 1.03	7.56 ± 0.96	7.50 ± 3.20	*p* = 0.220
Palmitic acid	C16:0	23.90 ± 2.44	20.78 ± 1.12	22.51 ± 1.08	20.49 ± 1.49	*p* = 0.065
Stearic acid	C18:0	7.80 ± 1.06	6.17 ± 0.98	6.03 ± 0.83	5.99 ± 1.25	*p* = 0.086
Palmitoleic acid	C16:1	2.45 ± 0.16	2.56 ± 0.64	2.63 ± 0.41	2.00 ± 0.79	*p* = 0.549
Oleic acid	C18:1	33.35 ± 2.71	33.65 ± 4.97	30.29 ± 2.53	32.05 ± 3.76	*p* = 0.405
Eicosenoic acid	C20:1n9	0.36 ± 0.06	0.37 ± 0.09	0.30 ± 0.02	0.36 ± 0.07	*p* = 0.436
Linoleic acid (LA)	C18:2n6	19.35 ± 2.88 ^a^	25.91 ±4.41 ^b^	25.26 ±5.03 ^b^	27.21 ± 5.02 ^b^	*p* = 0.041
γ-Linolenic acid (GLA)	C18:3n6	0.26 ± 0.06	0.25 ± 0.02	0.29 ± 0.06	0.29 ± 0.06	*p* = 0.700
Eicosatrienoic acid	C20:3n6	0.68 ± 0.13	0.78 ± 0.04	0.74 ± 0.07	0.78 ± 0.05	*p* = 0.226
Arachidonic acid (AA)	C20:4n6	0.74 ± 0.08	0.74 ± 0.13	0.84 ± 0.12	0.77 ± 0.09	*p* = 0.554
α-Linolenic acid (ALA)	C18:3n3	1.56 ± 0.21	1.62 ± 0.64	2.01 ± 0.51	1.30 ± 0.27	*p* = 0.257
Eicosatrienoic acid	C20:3n3	0.20 ± 0.04	0.17 ± 0.03	0.20 ± 0.03	0.20 ± 0.02	*p* = 0.401
Eicosatetraenoic acid	C20:4n3	0.18 ± 0.02	0.14 ± 0.02	0.16 ± 0.02	0.15 ± 0.01	*p* = 0.303
Eicosapentaenoic acid (EPA)	C20:5n3	0.27 ± 0.08	0.19 ± 0.03	0.25 ± 0.05	0.21 ± 0.03	*p* = 0.211
Docosapentaenoic acid	C22:5n3	0.37 ± 0.07	0.31 ± 0.05	0.42 ± 0.07	0.32 ± 0.04	*p* = 0.127
Docosahexaenoic acid (DHA)	C22:6n3	0.52 ± 0.09	0.42 ± 0.12	0.51 ± 0.16	0.38 ± 0.03	*p* = 0.386
	ΣSFA	39.71 ± 3.26 ^a^	32.91 ± 2.59 ^b^	36.09 ± 2.42 ^ab^	33.98 ± 5.23 ^ab^	*p* = 0.045
	ΣMUFA	36.16 ± 2.76	36.58 ± 4.65	33.23 ± 2.28	34.41 ± 4.57	*p* = 0.517
	ΣPUFA	24.13 ± 3.33 ^b^	30.51 ± 5.16 ^a^	30.68 ± 5.17 ^a^	31.61 ± 5.41 ^a^	*p* = 0.041
	Σn6-PUFA	21.03 ± 2.88 ^b^	27.68 ± 4.50 ^a^	27.13 ± 4.90 ^a^	29.05 ± 5.02 ^a^	*p* = 0.031
	Σn3-PUFA	3.10 ± 0.44	2.84 ± 0.69	3.55 ± 0.34	2.56 ± 0.39	*p* = 0.098
	n-6:n-3 PUFA	6.77 ± 0.07 ^c^	9.76 ± 1.31 ^b^	7.63 ± 1.06 ^c^	11.35 ± 0.50 ^a^	*p* < 0.001

Data are expressed as nmol% of the total extracted fatty acids; values are expressed as mean ± SD, *n* = 4–5. Data were analyzed by two-way ANOVA, and different superscripts (a, b, c) are used to denote significant differences between the treatment groups. Q—quartile; FA—fatty acids; ΣSFA—sum of saturated fatty acids; ΣMUFA—sum of monounsaturated fatty acids; ΣPUFA—sum of polyunsaturated fatty acids; Σn-6—sum of omega-6 polyunsaturated fatty acids; Σn-3—sum of omega-3 polyunsaturated fatty acids. *p* < 0.05 was considered significant.

## Data Availability

Not applicable.

## Supplementary Materials

The following supporting information can be downloaded at: https://www.mdpi.com/article/10.3390/biomedicines10051129/s1, Figure S1: CONSORT flow diagram for participant selection; Figure S2: Effects of breast milk from women with obesity on the mRNA expression of macrophage migration inhibitory factor (*Mif*); Figure S3: The effect of maternal obesity on malondialdehyde (MDA) levels in breast milk; Table S1: Primer sequences for qPCR; Table S2: The fatty acid composition of breast milk by quartiles with low and high n-6:n-3 PUFA ratios.
