# Supplementary material for: Breast Milk from Non-Obese Women with a High Omega-6 to Omega-3 Fatty Acid Ratio, but Not from Women with Obesity, Increases Lipogenic Gene Expression in 3T3-L1 Preadipocytes, Suggesting Adipocyte Dysfunction
Source: Biomedicines. 2022 May 13;10(5):1129. doi: 10.3390/biomedicines10051129 (PMC9138889; doi:10.3390/biomedicines10051129)
Supplement: Supplementary file 1 [file biomedicines-10-01129-s001.zip › biomedicines-1654057-supplementary.pdf]

Breast Milk from Non-Obese Women with a High Omega-6 to Omega-3 Fatty Acid Ratio, but Not from Women with Obesity, Increases Lipogenic Gene Expression in 3T3-L1 Preadipocytes, Suggesting Adipocyte Dysfunction

Peter Isesele [1], Samantha Enstad [2], Pham Huong [3], Raymond Thomas [3], Carol L. Wagner [4], Sarbattama Sen [5] and Sukhinder K. Cheema [1,*]

[1] Department of Biochemistry, Memorial University, St. John's, NL A1C 5S7, Canada; poisesele@mun.ca
[2] Winnie Palmer Hospital for Women and Babies, Orlando, FL 32806, USA; samantha.enstad@orlandohealth.com
[3] School of Science/Boreal Ecosystems and Agriculture Sciences, Memorial University, Corner Brook, NL A2H 5G4, Canada; tpham@grenfell.mun.ca (P.H.); rthomas@grenfell.mun.ca (R.T.)
[4] Department of Pediatrics, Division of Neonatology, Shawn Jenkins Children's Hospital, Medical University of South Carolina, Charleston, SC 29425, USA; wagnercl@musc.edu
[5] Department of Pediatric Newborn Medicine, Brigham and Women's Hospital and Harvard Medical School, Boston, MA 02115, USA; ssen2@bwh.harvard.edu
* Correspondence: skaur@mun.ca; Tel.: +17-09-864-3987

Keywords: adipogenesis; lipogenesis; breast milk; obesity; polyunsaturated fatty acids; 3T3-L1 preadipocytes

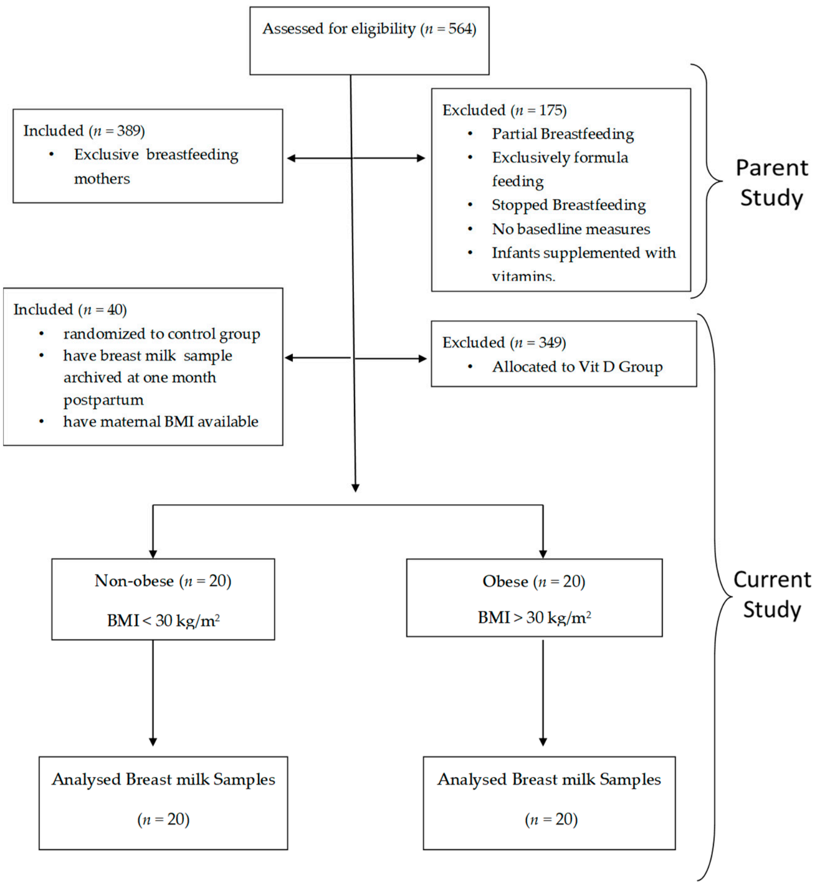

Figure S1: Consort flow diagnram for participant selection.

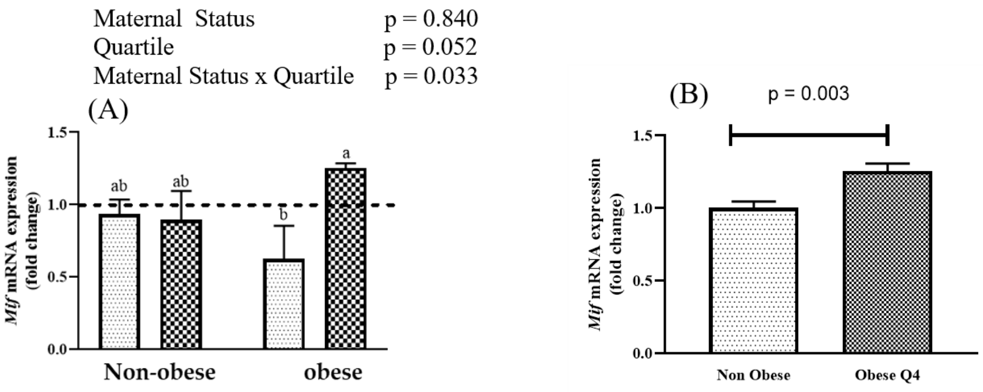

Figure S2. Effects of breast milk from women with obesity (obese) on the mRNA expression of Macrophage migration inhibitory factor (*Mif*). Values are expressed as mean ± SD, *n* = 3. Data were assessed using 2 way ANOVA in (A), and Student's t-test was used to analyze differences between control untreat cells (No BM) and obese Q4 (high n-6:n-3 PUFA) (B). P<0.05 was considered significant. Different superscripts (a, b) are used to denote significant differences between the treatment groups, BM = breast milk; Q = quartile.

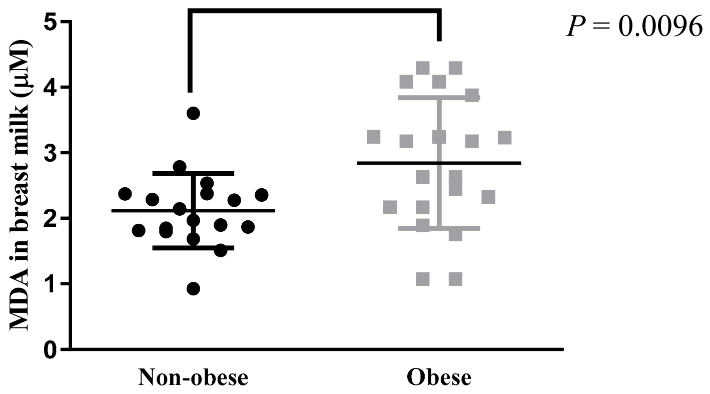

Figure S3. The effect of maternal obesity on malondialdehyde (MDA) levels in breast milk. MDA levels in the breast milk whey of non-obese and women with obesity (obese) at one month postpartum were measured using a Thiobarbituric acid reactive substances (TBARS) assay kit (KGE013, R & D Systems, Minnesota, USA) as previously described (Enstad et al., 2021). The pre-optical density readings were subtracted from the optical density values after 2 hours of incubation to get the final concentration and multiplied by the dilution factor to determine the MDA concentration in the samples. Data were expressed as micromole (µM), and the values are expressed as mean ± SD, *n* = 20. Data were analyzed using Student's t-test to determine the difference between the group; *P* <0.05 was considered significant.

Table S1. Primer sequences for qPCR.

Gene	Forward (5′-3′)	Reverse (5′-3′)
Acc1	GGCCAGTGCTATGCTGAGAT	AGGGTCAAGTGCTGCTCCA
Atgl	AACACCAGCATCCAGTTCAA	GAACTTGCCCATGTCCTTGT
Fasn	CTGCGGAAACTTCAGGAAATG	GGTTCGGAATGCTATCCAGG
Hsl	AGACACCAGCCAACGGAT	GGGCATAGTAGGCCATAGCA
Mif	TCAAGCGAAGGTGGAACCGTT	CGGACCGGGTCTACATCAA
Pparg	GAGCTGACCCAATGGTTGCTG	GCTTCAATCGGATGGTTCTTC
Plin1	TGCTGGATGGAGACCTC	ACCGGCTCCATGCTCCA
Rplp0	AATTTCAATGGTGCCTCTGG	TCACTGTGCCAGCTCAGAAC
Scd1	CACCTGCCTCTTCGGGATTT	CTTGACAGCCGGGTGTTTG

Acc1, acetyl-CoA carboxylase; *Atgl*, adipose triglyceride lipase; *Fasn*, fatty acid synthase; *Hsl*, hormone-sensitive lipase; *Mif,* macrophage migration inhibitory factor; *Plin1,* perilipin; *Pparg*, peroxisome proliferator-activated receptor-gamma; *Rplp0,* large ribosomal Protein; *Scd1*, stearoyl-CoA desaturase.

Table S2. The fatty acids composition of breast milk by quartiles with low and high n-6:n-3 PUFA.

	Non-obese				Obese			
FA (nmol%)	Q1	Q2	Q3	Q4	Q1	Q2	Q3	Q4
C14	7.14±1.74	6.13±1.18	6.28±1.65	5.38±1.03	6.75±0.97	5.27±0.97	7.59±3.30	6.78±3.20
C16	21.33±2.44	20.08±3.11	21.05±2.85	18.76±1.12	20.11±1.09	21.25±1.09	17.82±2.53	18.53±1.49
C18	6.96±1.06	6.38±1.26	5.46±0.51	5.57±0.98	5.38±0.83	4.20±0.83	4.62±0.48	5.42±1.25
C16:1	2.19±0.16	2.16±1.40	2.52±0.50	2.31±0.64	2.35±0.42	2.45±0.42	2.10±0.72	1.81±0.80
C18:1n9	27.07±2.44	27.58±0.99	25.83±2.65	27.81±4.63	24.38±2.00	21.81±2.00	23.50±3.86	26.73±3.37
C18:1n7	2.68±0.28	2.55±0.73	2.73±0.39	2.57±0.34	2.68±0.54	3.00±0.54	2.62±0.28	2.26±0.40
C20:1n9	0.33±0.06	0.31±0.04	0.31±0.07	0.33±0.09	0.27±0.03	0.27±0.03	0.29±0.08	0.33±0.08
C18:2n6	17.27±2.88	20.06±6.24	21.97±3.21	23.39±4.41	22.57±5.03	22.78±5.03	25.49±2.75	24.60±5.03
C18:3n6	0.23±0.06	0.25±0.04	0.22±0.04	0.22±0.02	0.26±0.06	0.20±0.06	0.25±0.05	0.26±0.07
C20:3n6	0.60±0.13	0.64±0.09	0.64±0.10	0.70±0.04	0.66±0.07	0.70±0.07	0.86±0.09	0.71±0.06
C20:4n6	0.66±0.08	0.69±0.11	0.68±0.14	0.67±0.13	0.75±0.13	0.61±0.13	0.71±0.10	0.70±0.10
C18:3n3	1.39±0.21	1.77±0.89	1.52±0.26	1.46±0.64	1.80±0.51	1.75±0.51	1.57±0.38	1.18±0.28
C20:3n3	0.17±0.04	0.17±0.01	0.21±0.05	0.15±0.03	0.18±0.03	0.14±0.03	0.21±0.06	0.18±0.02
C20:4n3	0.16±0.02	0.15±0.03	0.15±0.02	0.12±0.02	0.14±0.02	0.13±0.02	0.17±0.01	0.13±0.01
C20:5n3	0.24±0.08	0.20±0.04	0.23±0.05	0.17±0.03	0.23±0.06	0.14±0.06	0.21±0.03	0.19±0.04
C22:5n3	0.33±0.07	0.30±0.06	0.33±0.04	0.28±0.05	0.38±0.08	0.28±0.08	0.32±0.05	0.29±0.05
C22:6n3	0.47±0.09	0.40±0.17	0.42±0.09	0.38±0.12	0.45±0.17	0.30±0.17	0.33±0.04	0.34±0.04
n6-PUFA	18.77±2.88	21.63±6.28	23.52±3.15	24.99±4.50	24.23±4.91	24.29±4.91	27.32±2.80	26.27±5.03
n3-PUFA	2.77±0.44	2.99±0.90	2.86±0.35	2.56±0.69	3.18±0.34	2.73±0.34	2.81±0.36	2.31±0.39
n6/n3-PUFA	$6.77±0.07^{d}$	$7.25±0.32^{c}$	$8.23±0.38^{c}$	$9.76±1.31^{b}$	$7.60±1.06^{cd}$	$8.91±1.06^{b}$	$9.77±0.58^{b}$	$11.35±0.50^{a}$

Data were expressed as nmol% of the total extracted fatty acids; values are expressed as mean ± SD, n = 4-5. Data were analyzed by two-way ANOVA, and superscripts (a,b) were used to denote significant differences between the treatment group. Q= quartile, FA = fatty acids, ΣSFA = sum of saturated fatty acids, ΣMUFA= sum of monounsaturated fatty acids, ΣPUFA = sum of polyunsaturated fatty acids, Σn-6 sum of omega-6 polyunsaturated fatty acids, Σn-3 sum of omega-3 polyunsaturated fatty acids P<0.05 was considered significant.
